# Throat Swab Culture Positivity Rate, Antibiotic Susceptibility Profile, and Associated Risk Factors of *Streptococcus pyogen*es Among Children With Acute Pharyngitis Attending Jigjiga University Sheik Hassan Yebere Referral Hospital, Jigjiga, Ethiopia

**DOI:** 10.1155/ijm/8337012

**Published:** 2026-03-06

**Authors:** Surafel Mekuria, Fasil Getu, Muluken Walle, Adisu Tesfaye, Daniel Tolossa, Tigist Abebe, Zerihun Abera Ayele, Sara Tesfaye, Shamil Barsenga, Bawlah Tahir, Abdurehman Kedir Roble

**Affiliations:** ^1^ Department of Medical Laboratory Sciences, School of Medicine, Institute of Health Science, Jigjiga University, Jigjiga, Ethiopia, jju.edu.et; ^2^ Department of Hematology and Immunohematology, School of Biomedical and Laboratory Sciences, College of Medicine and Health Sciences, University of Gondar, Gondar, Ethiopia, uog.edu.et; ^3^ Department of Medicine, School of Medicine, Institute of Health Science, Jigjiga University, Jigjiga, Ethiopia, jju.edu.et; ^4^ Department of Midwifery, School of Nursing, Institute of Health Science, Jigjiga University, Jigjiga, Ethiopia, jju.edu.et

**Keywords:** Ethiopia, Jigjiga, pharyngitis, *Streptococcus pyogenes*

## Abstract

**Background:**

Pharyngitis is inflammation of the oropharynx primarily caused by *Streptococcus pyogen*es. *S. pyogenes* is a Gram‐positive, catalase‐negative, β‐hemolytic, bacitracin‐sensitive bacterium. Group A streptococci (GAS) pharyngitis causes the most outpatient visits and frequent antibiotic prescriptions among children. In the study area, there is a lack of studies on the prevalence and antimicrobial susceptibility pattern of GAS pharyngitis.

**Objective:**

To determine the throat swab culture positivity rate, antimicrobial resistance patterns, and associated factors of *S. pyogenes* infection among children with acute pharyngitis attending Jigjiga University Sheik Hassan Yebere Referral Hospital from February 8 to August 4, 2024, in Jigjiga, Ethiopia.

**Methodology:**

A cross‐sectional study was conducted among 468 participants recruited from the hospital pediatrics department through a convenience sampling method. Sociodemographic and clinical data were collected from the study participants using a structured questionnaire. Oropharyngeal swabs were collected and processed to identify the *S. pyogenes* pathogen using the conventional culture, followed by specific biochemical tests according to standard operating procedures. The Kirby–Bauer disk diffusion method was implemented for antimicrobial susceptibility testing. Data were entered into EpiData and then exported to SPSS Version 20, and the analysis was done.

**Result:**

Among the 468 study participants, 269 (57.5%) were males, and the mean age of the participants was 4, with a standard deviation (SD) of 3.6. The prevalence of *S. pyogenes* infection among children with pharyngitis attending the hospital was 14.3% (95% CI: 11.3–17.8). The presence of household smoking (AOR = 2.67; 95% CI: 1.47–4.89) and tonsillar swelling (AOR = 2.1; 95% CI: 1.03–4.19) were significantly associated with *S. pyogenes* infection. The isolated organisms showed resistance to tetracycline (17.9%), azithromycin (11.9%), chloramphenicol (10.4%), and clindamycin (9%).

**Conclusion:**

The findings indicate that the prevalence of *S. pyogenes* infection in this study was slightly higher than that in comparable studies. The presence of household smokers and of tonsillar swelling was significantly associated with the positive *S. pyogenes* diagnosis.

## 1. Introduction

Pharyngitis is the inflammation of the mucous membranes of the oropharynx, most commonly caused by bacterial or viral infections, although allergies and other factors may also contribute [1]. *Streptococcus pyogenes* (Group A streptococcus [GAS]) is a leading cause of acute pharyngitis, primarily affecting the oropharynx, posterior pharynx, and tonsils. It is also responsible for various poststreptococcal complications [2, 3]. This should be treated with antibiotics within 9 days from onset to eliminate the risk of rheumatic heart disease (RHD), and it is therefore important for patients to seek skilled health care when symptoms onset promptly [4]. Globally, an estimated 600 million symptomatic cases of GAS pharyngitis occur annually, with more than 550 million reported from low‐income countries [5]. GAS accounts for 20%–30% of pediatric pharyngitis cases and about 15% of cases in young adults [6].

*S. pyogenes* is a Gram‐positive, catalase‐negative, coagulase‐negative coccus that occurs in pairs or chains. The bacteria show β‐hemolytic streptococci when they grow on blood agar media. It is a facultative anaerobe that grows best in 5%–10% carbon dioxide and forms pinpoint colonies on media plates. The Lancefield serological grouping system is used to differentiate GAS from other streptococci [7]. *S. pyogenes* is a highly contagious bacterial organism that can be spread person to person through direct or indirect contact with respiratory secretions and wounds [8]. It is primarily transmitted from person to person through respiratory droplets, with a short incubation period of 2–5 days. GAS pharyngitis peaks in the late winter and early spring months when children are predominantly indoors for school and sports. *S. pyogenes* pharyngitis frequently impacts more children in low‐ and middle‐income countries due to overcrowding conditions [9].

Worldwide, GAS infections contribute significantly to morbidity, with millions of sore throat episodes in children and substantial disability‐adjusted life years (DALYs) each year [10]. The incidence of severe GAS disease is notably higher in sub‐Saharan Africa compared to high‐income countries, with a rate of 5 per 1000 in sub‐Saharan Africa but only 0.3 per 1000 in developed countries [11–13]. This infection has a critical place in developing countries’ health systems, touching on patient education and primary care, and requiring costly interventions if not prevented [14].

Despite its public health significance, data on the true burden of GAS pharyngitis in Africa remain limited [13]. Available studies indicate considerable variation across countries; the prevalence of GAS pharyngitis, with its burden and consequence, is generally higher in developing countries and impoverished communities than in industrialized nations [15]. Globally, there are approximately 1.78 million new cases of GAS infection each year [11]. In a cross‐sectional study conducted in India among a total of 218 throat swabs from patients with acute pharyngitis, the overall prevalence of GAS pharyngitis was 9.17% [16]. In a cross‐sectional study conducted at Jima town health centers among 355 children with pharyngitis, the prevalence of GAS pharyngitis was 11.3% [17]. Similarly, a cross‐sectional study conducted at Felege Hiwot Comprehensive Specialized Hospital, Northwest Ethiopia, reported a 9.1% prevalence of culture‐confirmed GAS pharyngitis among children attending the hospital [2]. Currently, antibiotic resistance among bacteria is increasing due to factors such as misuse and overuse of antibiotics, poor infection prevention practices, and illegal purchasing and use of antimicrobial treatments, leading to poor treatment outcomes in many communities [18]. In a cross‐sectional study conducted on 213 acute pharyngitis‐suspected pediatric patients from April to September 2022 at Hawassa University Comprehensive Specialized Hospital and Yirgalem Hospital among isolated *S. pyogenes* bacteria, all isolates were sensitive to penicillin and amoxicillin, but in contrast, 8 (36.4%) of isolates exhibited resistance to tetracycline, 7 (31.8%) to ceftriaxone, 6 (27.3%) to erythromycin, and 5 (22.7%) isolates showed drug resistance [19]. In a cross‐sectional study conducted at Felege Hiwot Comprehensive Specialized Hospital, Northwest Ethiopia, among all isolated GAS bacteria, antimicrobial drug resistance was observed as follows: ceftriaxone 35.7%, vancomycin 35.5%, erythromycin 21.4%, tetracycline 14.3%, chloramphenicol 7.1%, clindamycin 50%, and levofloxacin 7.1% [2].

In Ethiopia, particularly in the Somali region capital, Jigjiga, there is a lack of data available on the overall throat swab culture positivity rate of *S. pyogenes* and antibiotic drug resistance patterns among children under 18 years of age. There is limited knowledge in the community regarding the bacteria. Therefore, to fill this gap, this study was conducted to determine the overall prevalence, associated risk factors, and antibiotic drug resistance of *S. pyogenes* bacteria among pediatric patients with pharyngitis.

## 2. Materials and Methods

### 2.1. Study Area Period

The study was conducted in Jigjiga town at Sheik Hassan Yebere Referral Hospital from February 8 to August 4, 2024, in Jigjiga, Ethiopia. This referral hospital currently provides health services in Jigjiga town and accepts numerous referral cases from nearby cities of the Somali region. It is located in the Fafan zone, 60 km west of the border with Somalia and 625 km from the capital city of Ethiopia, Addis Ababa [20].

### 2.2. Study Design

A health facility‐based quantitative cross‐sectional study was employed.

### 2.3. Source Population

All children under 18 years of age with acute symptomatic pharyngitis who visited the pediatric outpatient department of Sheik Hassan Yebere Referral Hospital were included.

### 2.4. Study Subjects

All selected pediatric patients with symptomatic pharyngitis who visited the pediatrics outpatient department of Sheik Hassan Yebere Referral Hospital during the study period.

### 2.5. Eligibility Criteria

All symptomatic children’s patients with acute pharyngitis who visited the pediatric OPD and could give the required sample were included, but children who were already on antibiotic treatment were excluded from the study to prevent laboratory false result errors.

### 2.6. Sample Size Determination

The sample size was determined using a double population proportion formula to calculate the required sample size for GAS pharyngitis, considering a lower age group [21] as the major determinant factor of *S. pyogenes* infection with its adjusted odds ratio (AOR) and by using a power of 80% and confidence interval (CI) of 95% by using EPI INFO Stat Calculator Version 7 software. Finally, a 10% nonresponse rate was added, and the overall sample size was 468 (see Table 1).

**TABLE 1:** Sample size determination for the second objective by the two‐population proportion formula.

Variables	Confidence interval	Exposed (%)	Unexposed (%)	AOR	Ratio	Sample size
Lower age	95%	15.4	4.8	3.34	1:1	425

*Note:* 425 + 10% nonresponse rate = 468.

### 2.7. Sampling Procedure

In Jigjiga town, there are three hospitals; among them, the referral hospital was selected using a simple random sampling method, and study participants were then included using a convenience sampling method.

### 2.8. Data Collection and Laboratory Processing

All relevant data, like sociodemographic and clinical data, were collected by trained data collectors, including two BSC nurses and two laboratory technicians. Firstly, after obtaining written informed consent from the child’s parent or guardian, data were collected through face‐to‐face interviews using a semistructured questionnaire administered to the parent or guardian. The clinical sample (throat swab) was collected and placed in Amies transport medium, maintained at a temperature of 2–8°C using an ice pack, and transported to the microbiology laboratory of Sheik Hassan Yebere Referral Hospital.

### 2.9. Culturing and Identification of the Bacteria

Upon arrival, the specimens were cultured at Jigjiga University Sheik Hassan Yebere Referral Hospital Microbiology laboratory for the isolation of *S. pyogenes* (GAS). The sample obtained was inoculated on blood agar supplemented with 5% sheep blood. Then the plates were incubated at 37°C with 5% CO_2_ for 18–24 h using strict standard operating procedures (SOPs). Colonies that showed beta hemolysis (complete zone of inhibition) on blood agar were further identified by biochemical tests, including the catalase test and sensitivity to the bacitracin disk, indicated by a zone of inhibition of ≥ 10 mm. Representative colonies suspected to be *S. pyogenes* bacteria were selected based on strict criteria, and the streptococci were examined microscopically using a 100x oil immersion objective [22].

Antimicrobial sensitivity testing (AST): AST was done on identified *S*. *pyogenes* bacterial pathogens by using the disk diffusion method on Mueller–Hinton agar (MHA) supplemented with 5% sheep blood. Colony suspension was prepared by mixing it with normal saline (0.85% NaCl), which made it equivalent to a 0.5% McFarland standard. Before conducting the AST, we obtained information from the physician on antibacterials commonly ordered to treat *S. pyogenes* bacteria. Then those antimicrobials were compared with the Clinical and Laboratory Standards Institute (CLSI) guidelines [23]. The antimicrobial agents tested were penicillin (10 U), ceftriaxone (30 μg), azithromycin (15 μg), tetracycline (30 μg), chloramphenicol (30 μg), clindamycin (2 μg), and vancomycin (30 μg). Antimicrobial susceptibility testing was performed on MHA supplemented with 5% sheep blood using the Kirby–Bauer disk diffusion method. The zone of inhibition was measured using a ruler, recorded, and then compared with CLSI standards using the appropriate technique [23].

### 2.10. Data Quality Control

The questionnaire was translated into commonly used languages in the study area, namely, Somali and Amharic, and back‐translated into English to ensure consistency. The principal investigator provided training to data collectors on data collection, sample collection, and transportation procedures. A pretest was conducted on 5% of the sample size in a nonstudy area at Karamara General Hospital before the actual data collection, and the pretest data were excluded from the final analysis. The quality of the prepared media was checked by incubating it for 24 h at 37°C without inoculation to check the presence of contaminants, and then for the drug susceptibility test, quality control strains of *S*. *pyogenes* American Type Culture Control (ATCC) 12344 were used to check proper bacterial growth characteristics. The zone of inhibition in diameters obtained for the control strain was compared with the CLSI [23].

The bacitracin disk was checked by using positive and negative controls. *S*. *pyogenes* ATCC 12344 and *Staphylococcus aureus* ATCC 25953, respectively. The supervisor monitored the overall progress of the research throughout the study period.

### 2.11. Data Processing and Analysis

The data were coded and entered in EpiData Version 3.1 upon creating the questionnaire template in the software. The entered data were cleaned to ensure the validity of the data. The prepared data were exported and analyzed using the Statistical Package for the Social Sciences (SPSS), Version 20. Descriptive statistics, including frequencies, proportions, means, and SDs, were calculated for the independent. An AOR was used to assess the association and statistical significance between independent and dependent variables. All variables were analyzed in the bivariate logistic regression, and a variable with a *p* value ≤ 0.25 was further considered for multivariate logistic regression to control for confounding variables. An AOR with a 95% CI and *p* value ≤ 0.05 was considered statistically significant. Finally, the data were subsequently presented in tables and figures using SPSS Version 20 and the Microsoft Excel Office 365 program.

### 2.12. Ethical Consideration

Ethical clearance was obtained from the Institutional Health Research Ethics Review Committee of Jigjiga University (Reference No. *JJU RERC 061/2024*), in accordance with the Declaration of Helsinki. A formal support letter was provided to Sheik Hassan Yebere Referral Hospital to secure institutional permission.

Written informed consent was obtained from the parent or legal guardians of all participating children after explaining the purpose, procedures, benefits, and possible risks of the study. They were informed that participation was voluntary, refusal carried no penalty, and they could withdraw at any time. Confidentiality of all collected information and laboratory results was strictly maintained. Children who tested positive for *S. pyogenes* were referred to the attending physician for appropriate treatment.

## 3. Results

### 3.1. Sociodemographic Characteristics of the Respondents

Four hundred sixty‐eight children aged less than 18 years with acute pharyngitis were included in the study, with a 100% response rate. Among 468 children’s patients, 269 (57.5%) were males. The highest proportion of pediatric patients was in the (0–4) year age group (67.1%). The mean age of the study participants was 4 years with an SD of 3.6. Most study participants, 386 (82.5%), were of Somali ethnicity. Regarding parental education, the largest proportion of parents, 260 (55.6%), had elementary‐level education, while 22 (4.7%) had attained higher education (Table 2).

**TABLE 2:** Sociodemographic characteristics of pediatric patients attending Sheik Hasan Yebere Referral Hospital, Jigjiga, Ethiopia, from February 8 to August 4, 2024.

Variables	Category	Frequency	Percent
Age	0–4	332	70.9
	5–9	86	18.4
	≥ 10	50	10.7

Sex	Male	269	57.5
	Female	199	42.5

Religion	Muslim	392	83.8
	Orthodox	57	12.2
	Protestant	18	3.8
	Catholic	1	0.2

Residence	Rural	93	19.9
	Urban	375	80.1

Ethnicity	Somali	386	82.5
	Amhara	46	9.8
	Oromo	28	6.0
	Gurage	8	1.7

Educational status of the mother	Not read/write	73	15.6
	Primary	260	55.6
	Secondary	113	24.1
	Diploma and above	22	4.7

Marital status	Married	365	78.0
	Widowed	17	3.6
	Divorced	27	5.8
	Single	59	12.6

Mothers’ occupation	Housewife	244	52.1
	Government worker	145	31
	Merchant	52	11.1
	Others	27	5.8

### 3.2. Income and Living Conditions

Among all study participants, the highest proportion of family members, 295 (63%) lived with a monthly income of greater than or equal to 10,000 ETB, and from all participants, 101 (21.6%) children lived in a family with a member who was a cigarette or shisha smoker, and the vast majority, 256 (54.7%), of study participants lived with a family member with greater than 5 members (Table 3).

**TABLE 3:** Income and living conditions of pediatric patients attending Sheik Hassan Yebere Referral Hospital, Jigjiga, Ethiopia, from February 8 to August 4, 2024.

Variables	Category	Frequency	Percent
Monthly income of the family	< 10,000 ETB	173	37
	> 10,000 ETB	295	63

Smoker availability in the house	Yes	101	21.6
	No	367	78.4

No. of family members	> 5	256	54.7
	< 5	212	45.3

### 3.3. Medical History and Comorbid Conditions

Among all child study participants, 332 (70.9%) had fever, 317 (67.7%) had tonsillar swelling, 203 (43.4%) had cough, and 182 (38.9%) had dysphagia during the study period (Table 4).

**TABLE 4:** Medical histories of pediatric patients attending Sheik Hasan Yebere Referral Hospital, Jigjiga, Ethiopia, from February 8 to August 4, 2024.

Variables	Category	Frequency	Percent
Fever	Yes	332	70.9
	No	136	29.1

Tonsillar swelling	Yes	317	67.7
	No	151	32.3

Rash	Yes	108	23.1
	No	360	76.9

Cough	Yes	203	43.4
	No	265	56.6

Dysphagia	Yes	182	38.9
	No	286	61.1

BMI	Underweight	94	20.1
	Overweight	25	5.3
	Normal	349	74.6

### 3.4. Prevalence of *S. pyogenes* Among Pediatric Patients With Pharyngitis

The prevalence of throat swab culture positive for *S*. *pyogenes* (GAS) bacterial infection among pediatric patients attending Sheik Hassan Yebere Hospital was 67/468 (14.3%) (95% CI: 11.3–17.8).

### 3.5. Factors Associated With the Presence of *S*. *pyogenes* (GAS) Infection Among Pharyngitis Patients

Factors such as family size, the presence of smokers in the household, cough, and tonsillar swelling showed a significant association in the binary logistic regression analysis. These variables were subsequently included in the multiple logistic regression model to control for potential confounding factors (Table 5).

**TABLE 5:** Bivariate analyses for factors associated with *S. pyogenes* (GAS) infection among pediatric patients with acute pharyngitis attending Sheik Hasan Yebere Referral Hospital, Jigjiga, Ethiopia, from February 8 to August 4, 2024.

Variables	Category	*Streptococcus pyogenes* (GAS)		Bivariate analysis	
		Positive *n* (%)	Negative *n* (%)	COR (95% CI)	*p* value
Sex	Male	42 (15.6%)	227 (84.3%)	1.3 (0.75–2.2)	0.35
	Female	25 (12.5%)	174 (87.4%)	1	

Age	0–4	51 (15.4%)	281 (84.6%)	1.6 (0.6–4.3)	0.32
	5–9	11 (12.8%)	75 (87.2%)	1.3 (0.43–4.04)	0.63
	≥ 10	5 (10%)	45 (90%)	1	

Residence	Rural	18 (19.4%)	75 (80.6%)	1.6 (0.8–2.9)	0.13
	Urban	49 (8.3%)	326 (91.7%)	1	

Parent marital status	Single	5 (8.5%)	54 (91.5%)	0.5 (0.19–1.31)	0.23
	Divorced	1 (3.7%)	26 (96.3%)	0.21 (0.03–1.56)	0.13
	Widowed	4 (23.5%)	13 (76.5%)	1.6 (0.58–5.2)	0.39
	Married	57 (15.6%)	308 (84.3%)	1	

Parent educational status	Not read/write	20 (27.4%)	53 (72.6%)	7.9 (0.9–62.8)	0.05
	Elementary	31 (11.9%)	229 (88.1%)	2.8 (0.37–21.9)	0.31
	Secondary	15 (13.3)	98 (86.7)	3.2 (0.40–25.6)	0.27
	Diploma/above	1 (4.5%)	21 (95.5)	1	

Mother’s occupation	Housewife	42 (17.2%)	202 (82.7%)	1	
	Government worker	15 (10.3%)	130 (89.7%)	0.55 (0.29–1.1)	0.06
	Merchant	6 (11.5%)	46 (88.4%)	0.63 (0.25–1.6)	0.31
	Others	4 (14.8%)	23 (85.2%)	0.8 (0.27–2.54)	0.75

The monthly income of the parent	< 10,000 ETB	27 (15.6%)	146 (84.4%)	1.17 (0.69–2.00)	0.54
	> 10,000 ETB	40 (13.6%)	255 (86.4%)	1	

No. of family members	**> 5**	**47 (18.4%)**	**209 (81.6%)**	**2.16 (1.24–3.77)**	**0.007**
	< 5	20 (9.4%)	192 (90.6%)	1	

Smoker availability in the house	Yes	**28 (27.7%)**	**73 (72.3)**	**3.23 (1.86–5.58)**	**0.001**
	No	39 (10.6%)	328 (89.4%)	1	

Fever	Yes	51 (15.4%)	281 (84.6%)	1.36 (0.74–2.48)	0.31
	No	16 (11.8%)	120 (88.2%)	1	

Tonsillar swelling	Yes	**55 (17.4%)**	**262 (82.6%)**	**2.43 (1.26–4.69)**	**0.01**
	No	12 (7.9%)	139 (92.1%)	1	

Rash	Yes	14 (13%)	94 (87%)	0.86 (0.45–1.6)	0.65
	No	53 (14.7%)	307 (85.3%)	1	

Cough	Yes	**40 (19.7%)**	**163 (80.3%)**	**2.16 (1.27–3.67)**	**0.004**
	No	27 (10.2%)	238 (89.8)	1	

Dysphagia	Yes	30 (16.5%)	152 (83.5%)	1.33 (0.78–2.23)	0.28
	No	37 (12.9%)	249 (87.1%)	1	

BMI	Underweight	11 (11.7%)	83 (88.3%)	0.74 (0.37–1.40)	0.39
	Overweight	3 (12%)	22 (88%)	0.76 (0.22–2.63)	0.66
	Normal	53 (15.2%)	296 (84.8%)	1	

*Note:* Variables which showed significant association with S. pyogenes (GAS) bacterial infection.

Variables like the number of family members, smokers’ availability in the house, cough, and tonsillar swelling were analyzed by multivariate analysis, and then those children patients participants who were living in a house having a cigarette or shisha smoker were 2.67 times more likely to develop GAS pharyngitis infection as compared to those who lived with nonsmokers (AOR = 2.67; 95% CI 1.47–4.89; *p* value ≤ 0.001). Finally, study participants with tonsillar swelling were 2.1 times more likely to develop GAS pharyngitis infection than those without swelling (AOR = 2.1; 95% CI: 1.03–4.19; *p* value 0.01) (Table 6).

**TABLE 6:** Multivariable analyses for factors associated with *Streptococcus pyogenes* (GAS) infection among pediatric patients with acute pharyngitis attending Sheik Hasan Yebere Referral Hospital, Jigjiga, Ethiopia, from February 8 to August 4, 2024.

Variables	Category	*Streptococcus pyogenes* (GAS)		Bivariate analysis		Multivariable analysis	
		Positive *n* (%)	Negative *n* (%)	COR (95% CI)	*p* value	AOR (95% CI)	*p* value
No. of family members	> 5	47 (18.4%)	209 (81.6%)	2.16 (1.24–3.77)	0.007	1.69 (0.89–3.17)	0.1
	< 5	20 (9.4%)	192 (90.6%)	1		1	

Smoker availability in the house	**Yes**	**28 (27.7%)**	**73 (72.3)**	**3.23 (1.86–5.58)**	**0.001**	2.67 (1.47‐4.89)**^∗^**	**0.001**
	No	39 (10.6%)	328 (89.4%)	1		1	

Tonsillar swelling	**Yes**	**55 (17.4%)**	**262 (82.6%)**	**2.43 (1.26–4.69)**	**0.01**	2.1 (1.03‐4.19)**^∗^**	**0.04**
	No	12 (7.9%)	139 (92.1%)	1		1	

Cough	Yes	40 (19.7%)	163 (80.3%)	2.16 (1.27–3.67)	0.004	1.4 (0.78–2.61)	0.24
	No	27 (10.2%)	238 (89.8)	1		1	

^∗^Variables that showed significant association with *S. pyogenes* (GAS) bacterial infection (*p* value < 0.05).

### 3.6. Antimicrobial Drug Susceptibility Testing

Among the isolated *S. pyogenes* bacteria, resistance was observed to several antibiotics: tetracycline was 12 (17.9%), azithromycin 8 (11.9%), chloramphenicol 7 (10.4%), clindamycin 6 (9%), ceftriaxone 5 (7.5%), and vancomycin 3 (4.5%) (Table 7). Regarding the multidrug resistance pattern, 11 isolates (16.4%) were resistant to two antibiotics, while 4 isolates (6%) showed resistance to three or more antibiotics (Table 8).

**TABLE 7:** Antimicrobial susceptibility pattern of *S. pyogenes* isolated from acute pharyngitis pediatric patients who attended pediatric Sheik Hassan Yebere Referral Hospital, Jigjiga, Ethiopia, from February 8 to August 4, 2024.

Antibiotic	Sensitive	Resistant	Intermediate
Penicillin	67 (100%)	—	—
Ceftriaxone	62 (92.5%)	5 (7.5%)	—
Azithromycin	57 (85.1%)	8 (11.9%)	2 (3%)
Tetracycline	48 (71.6%)	12 (17.9%)	7 (10.4%)
Chloramphenicol	59 (88.1%)	7 (10.4%)	1 (1.5%)
Clindamycin	61 (91%)	6 (9%)	—
Vancomycin	64 (95.5%)	3 (4.5%)	—

**TABLE 8:** Multidrug‐resistant pattern of *S. pyogenes* isolated from acute pharyngitis in pediatric patients who attended the pediatric Sheik Hassan Yebere Referral Hospital, Jigjiga, Ethiopia, from February 8 to August 4, 2024.

Antibiotics	Frequency
Ceftriaxone + azithromycin	2
Tetracycline + azithromycin	4
Tetracycline + chloramphenicol	3
Clindamycin + chloramphenicol	2
Tetracycline + vancomycin + chloramphenicol	1
Vancomycin + clindamycin + chloramphenicol	1
Vancomycin + tetracycline + azithromycin + ceftriaxone	2

## 4. Discussion

Pharyngitis is the inflammation of the oropharynx and mucous membranes of the oral cavity. It may be caused by bacteria or other microorganisms [1]. *S. pyogenes* is a Gram‐positive bacterium responsible for acute pharyngitis [24]. In this study, the prevalence of *S. pyogenes* infection was 14.3%, but this study has a lower prevalence than the other study done by systematic review, with the prevalence rate of GAS pharyngitis of 37% and 24% in the other two different studies [25, 26]. This difference may be attributed to the detection method, as the latter two studies used serologically confirmed diagnoses, which may have increased the reported prevalence. In the other descriptive cross‐sectional study conducted at KNH PEU, the overall GAS pharyngitis rate was 38.4%, which was higher than our study finding [27]. This difference may be due to the use of both culture methods and additional serological identification in the latter study, which may have increased the detection rate. The other cross‐sectional study done in Iran had a prevalence of 30%, which was higher than our study area [28]. This difference may be attributed to study duration, as the Iranian study was conducted over 2 years, which is longer than the duration of the present study, and may have contributed to the higher prevalence rate.

The other cross‐sectional study conducted in Yemen among pharyngitis‐confirmed GAS infections, the prevalence was 41.5%, which was greater than in our study area [29]. This difference may be due to the use of both rapid GAS antigen detection tests (RADTs) and gene‐based detection methods in the Yemen study, which may have increased the detection rate. Similarly, a case–control study conducted in Egypt among acute pharyngitis (cases) reported an overall GAS pharyngitis prevalence of 42.2%, which is higher than that observed in our study area. [30]. This difference may reflect variations in the study design, as the Egyptian study used a case–control approach with additional serologic detection methods.

A study conducted at Naresuan University Hospital, Phitsanulok, Thailand, found that the overall culture positivity for GAS pharyngitis was 7% [31]. The result was lower than that reported in this study, which may be due to geographical differences. In another cross‐sectional study conducted in a primary healthcare center in Fez, Morocco, the prevalence of GAS pharyngitis was 6.2% [32]. This result was less than that of our study area. This difference may be due to variations in geographical setting and study site; the Moroccan study was conducted in primary health care facilities, which may have resulted in a lower prevalence rate.

In the other cross‐sectional study conducted in Jima town, the prevalence of GAS pharyngitis was 11.3%, which was a little less than this finding [17]. This difference could be a geographical difference. Similarly, the other cross‐sectional study carried out on the overall prevalence of GAS bacterial infection was 11.8% [33]. This difference may be due to the sampling method used; the Nigerian study employed multistage sampling, which differs from the sampling method used in this study. Another hospital‐based cross‐sectional study conducted at the University of Gondar Comprehensive Specialized Hospital among children with acute pharyngitis reported a prevalence of *S. pyogenes* (GAS) of 10.7%, which is lower than the prevalence observed in this study [34]. The difference could be the study area difference; the Gonder study was conducted in the highland area, but this study was in the lowland part of the Ethiopian region. A cross‐sectional study conducted at three government health institutions in Arba Minch found that the prevalence of GAS pharyngitis was 8.8% [35]. The difference could be due to variations in the geographical setting and the living standard. A cross‐sectional study design conducted at Hawassa, South Ethiopia, among school‐aged children, the prevalence of throat swab GAS pharyngitis infection was 12.2% [21]. The result was less than this research finding. The difference may be due to the participant age inclusion, which means the Hawasa study included only school‐age children, which may have contributed to a lower prevalence rate.

In this study, several factors were significantly associated with the occurrence of GAS pharyngitis; among these factors, the presence of a smoker in the household was a significant factor. Children living in households with cigarette or shisha smokers were 2.67 times more likely to develop *S. pyogenes* infection compared with those living in nonsmoking households. This was less than the study conducted in Deberebrihan children, who lived in a smoker family and had an increased risk of GAS pharyngitis infection with an AOR of   4.87 [36]. This difference could be due to geographical and climatic differences, which means the Debrebrihan area is a colder region. Another hospital‐based cross‐sectional study design conducted at Limbe Regional Hospital, South West, Cameroon, among pediatric patients, found that children whose family members had no history of smoking were 86% protected from the occurrence of *S. pyogenes* pharyngitis compared to those who lived in a household with smokers [37].

In this research, children with tonsillar swelling were 2.1 times more likely to develop *S. pyogenes* infection than those who did not have it. This finding was less than that of the cross‐sectional study done in Jima town health center; children with tonsillar swelling had a 4.48 times risk of GAS pharyngitis [17]. This difference may be due to variation in living conditions and study setting, as the Jima study was conducted at the health center level. In another descriptive cross‐sectional study conducted at KNH PEU among 198 children with acute pharyngitis, an inflamed pharynx was found to be associated with GAS infection, with an AOR of 1.9 [27]. This finding was less than our study’s finding. The difference could be a big difference in the sampling size. Similarly, our study findings are also less than the study done at three government health institutions in Arba Minch; the history of tonsillar swelling was significantly associated with the occurrence of GAS pharyngitis, with an AOR of 5.1 [21]. This variation may be due to differences in study site selection, as the Arbaminch study was conducted across three governmental institutions, which could have contributed to the higher reported results.

Regarding drug resistance, the isolated *S. pyogenes* bacteria showed drug resistance to commonly used antimicrobials. The antibiotics selected for testing were based on CLSI guidelines and cross‐checked with frequently prescribed drugs. The observed resistance rates were 17.9% for tetracycline, 11.9% for azithromycin, 10.4% for chloramphenicol, 9% for clindamycin, 7.5% for ceftriaxone, and 4.5% for vancomycin. The findings were compared with various studies conducted worldwide. A cross‐sectional study conducted in Iran revealed that isolated *S. pyogenes* bacteria showed antibiotic resistance of 50.8% to azithromycin, 37.2% to clarithromycin, and 15.2% to clindamycin [28]. Regarding this finding, the results of azithromycin and clindamycin have lower resistance than those of our study. The difference might be a geographical difference. The finding of this study regarding resistance to tetracycline is a lower resistance rate compared to the study result at Naresuan University Hospital, Phitsanulok Province, which had a resistance rate of 70% [31]. Similarly, another study conducted in Jima showed a higher resistance rate to tetracycline than our study result, which showed a resistance rate of 52.5% [17]. In the other study conducted at Limbe Regional Hospital, South West, Cameroon, among pediatric patients with pharyngitis, the *S. pyogenes* isolated organisms showed antimicrobial resistance for clindamycin 50%, ceftriaxone 40%, chloramphenicol 20%, and tetracycline 20% [37]. When this result is compared with the study conducted from our study, it has had a higher resistance rate; this difference could be due to geographical area differences. In the other study conducted at Felege Hiwot Comprehensive Specialized Hospital, Northwest Ethiopia, among all isolated GAS bacteria, antimicrobial drug resistance was observed: ceftriaxone 35.7%, vancomycin 35.5%, erythromycin 21.4%, tetracycline 14.3%, chloramphenicol 7.1%, and clindamycin 50% [2]. Compared with our study, these findings showed higher resistance to antimicrobials such as ceftriaxone, clindamycin, and vancomycin, but lower resistance to tetracycline. These variations may be attributed to differences in geographical location, which can influence patterns of antibiotic use.

In the other study conducted in Arba Minch among all isolated GAS bacteria from pharyngitis patients, antibiotic drug resistance was observed to tetracycline 60% [21]. These results were higher than those observed in our study. The difference might be the Arbamich study, which was conducted in different public hospitals, whereas our study was conducted in a single hospital. Similarly, another cross‐sectional study conducted at the University Comprehensive Specialized Hospital and Yirgalem Hospital reported that among the isolated *S. pyogenes* bacteria, 8 (36.4%) showed resistance to tetracycline and 7 (31.8%) to ceftriaxone. These resistance rates were higher than those observed in our study. The variation may be due to differences in geographical setting, as the study was conducted in two separate nearby towns, which could have contributed to an increased resistance rate compared to our findings.

### 4.1. Limitations of the Study

The study employed a convenience sampling method that may limit the generalizability of the findings. This nonprobability approach was chosen due to feasibility constraints, including the unpredictable flow of patients, limited data collection time, and resource limitations that prevented the use of probability‐based sampling techniques.

The gold standard for identifying *S. pyogenes* is Lancefield grouping (e.g., detecting Group A antigen using latex agglutination). However, due to financial constraints and the shortage of latex agglutination reagents in my locality, the study relied solely on bacitracin sensitivity.

## 5. Conclusion

The prevalence and antimicrobial susceptibility pattern of the isolated *S. pyogenes* bacteria among pharyngitis patients in this study were slightly higher than those reported in similar research. The overall prevalence of *S. pyogenes* among children with acute pharyngitis attending Sheik Hassan Yebere Hospital was 14.3% (67/468). The presence of smokers in the household and visible tonsillar swelling were associated with a positive diagnosis of *S. pyogenes* infection. Among all *S. pyogenes* isolated bacteria, notable antibiotic resistance was observed, particularly to tetracycline 12 (17.9%) and azithromycin 8 (11.9%). Regarding multidrug resistance characteristics, 11 (16.4%) isolates were resistant to two antibiotics, while 4 (6%) isolates showed resistance to three or four antibiotics.

**Nomenclature**
AOR: Adjusted odds ratio
ATCC: American Type Culture Control
CI: Confidence interval
CLSI: Clinical Laboratory Standard Institute
DALYs: Disability‐adjusted life years
GAS: Group A streptococci
OPD: Outpatient department
RHD: Rheumatic heart disease
SOP: Standard operating procedure
SPSS: Statistical Package for Social Sciences

## Author Contributions

All authors contributed to this study, including conception, drafting, study design, data analysis, interpretation, and revision of the manuscript.

## Funding

The funds to conduct this research were fully covered by Jigjiga University.

## Disclosure

All authors approved the final version for publication and consented to its submission to the journal.

## Conflicts of Interest

The authors declare no conflicts of interest.

## Data Availability

The data that support the findings of this study are available from the corresponding author upon reasonable request.
